# Tracking social contact networks with online respondent-driven detection: who recruits whom?

**DOI:** 10.1186/s12879-015-1250-z

**Published:** 2015-11-14

**Authors:** Mart L. Stein, Peter G. M. van der Heijden, Vincent Buskens, Jim E. van Steenbergen, Linus Bengtsson, Carl E. Koppeschaar, Anna Thorson, Mirjam E. E. Kretzschmar

**Affiliations:** Julius Center for Health Sciences and Primary Care, University Medical Center Utrecht, Utrecht, The Netherlands; Centre for Infectious Disease Control, National Institute for Public Health and the Environment, Bilthoven, The Netherlands; Department of Methodology and Statistics, Faculty of Social and Behavioural Sciences, University Utrecht, Utrecht, The Netherlands; Southampton Statistical Sciences Research Institute, University of Southampton, Southampton, UK; Department of Sociology, Faculty of Social and Behavioural Sciences, University Utrecht, Utrecht, The Netherlands; Centre of Infectious Diseases, Leiden University Medical Centre, Leiden, The Netherlands; Department of Public Health Sciences-Global Health, Karolinska Institutet, Stockholm, Sweden; Flowminder Foundation, Stockholm, Sweden; Science in Action BV, Amsterdam, The Netherlands

**Keywords:** Social contact networks, Infectious diseases, Close-contact transmission, Respiratory pathogens, Disease outbreaks, Online survey methods

## Abstract

**Background:**

Transmission of respiratory pathogens in a population depends on the contact network patterns of individuals. To accurately understand and explain epidemic behaviour information on contact networks is required, but only limited empirical data is available. Online respondent-driven detection can provide relevant epidemiological data on numbers of contact persons and dynamics of contacts between pairs of individuals. We aimed to analyse contact networks with respect to sociodemographic and geographical characteristics, vaccine-induced immunity and self-reported symptoms.

**Methods:**

In 2014, volunteers from two large participatory surveillance panels in the Netherlands and Belgium were invited for a survey. Participants were asked to record numbers of contacts at different locations and self-reported influenza-like-illness symptoms, and to invite 4 individuals they had met face to face in the preceding 2 weeks. We calculated correlations between linked individuals to investigate mixing patterns.

**Results:**

In total 1560 individuals completed the survey who reported in total 30591 contact persons; 488 recruiter-recruit pairs were analysed. Recruitment was assortative by age, education, household size, influenza vaccination status and sentiments, indicating that participants tended to recruit contact persons similar to themselves. We also found assortative recruitment by symptoms, reaffirming our objective of sampling contact persons whom a participant may infect or by whom a participant may get infected in case of an outbreak. Recruitment was random by sex and numbers of contact persons. Relationships between pairs were influenced by the spatial distribution of peer recruitment.

**Conclusions:**

Although complex mechanisms influence online peer recruitment, the observed statistical relationships reflected the observed contact network patterns in the general population relevant for the transmission of respiratory pathogens. This provides useful and innovative input for predictive epidemic models relying on network information.

**Electronic supplementary material:**

The online version of this article (doi:10.1186/s12879-015-1250-z) contains supplementary material, which is available to authorized users.

## Background

For infectious diseases, such as influenza, severe acute respiratory syndrome and measles, proximity and social contact between individuals are major factors for person-to-person transmission. Knowledge on contact patterns is therefore important for the design of optimal outbreak control strategies [1–4]. To accurately understand and explain epidemic dynamics, information is required on the underlying contact network of a host population, i.e., a network that contains all contact persons potentially at risk for infection. For example, the number of contacts an infectious individual has with susceptible persons determines among others the basic reproduction number R_0_ (i.e., the number of secondary cases one case generates in a susceptible population) [5].

Contact networks are complex and highly dynamic (i.e., not constant over time) [6]. Previous empirical studies of contact patterns used different methods of data collection, including direct observation, contact diaries and electronic proximity sensors, to quantify social mixing behaviour for a variety of populations [7, 8]. For example, the POLYMOD study, a large randomized study in eight European countries, used contact diaries to analyse mixing patterns of independent respondents [9]. Despite controversies on the different modes of transmission of respiratory infectious diseases [10], face-to-face conversations and physical contact are often used as proxies for potential infectious contacts [9, 11]. Close contact persons such as family, friends and colleagues are thereby assumed to capture the majority of contacts for potential transmission events [12].

A social network approach can provide relevant epidemiological data on numbers of contacts and the strength and dynamics of contacts between pairs of individuals in a population [13, 14]. Respondent-driven detection, a method of detection derived from snowball sampling, is a chain recruitment method that allows for systematic sampling of contact persons of participants. Previously, we demonstrated that under certain conditions such a recruitment method can be applied online to extract topological properties of contact networks in an anonymous manner [15, 16]. This approach provides novel insights in contact network structures compared to studies that sampled participants independently of one another and collected no information about the network beyond the contact persons reported by participants [7]. In these earlier studies ‘seed’ individuals of similar age groups and backgrounds were sampled at convenience [15, 16]. Furthermore, complex mechanisms may play a role when participants choose from amongst their contact persons and when contact persons decide whether to join the survey [11]. For example, with an offline (i.e., paper based) chain recruitment method participants have a tendency to recruit spatially proximal peers [17]. This determines the type of contact networks being sampled. Note that we distinguish respondent-driven detection from respondent-driven sampling as our main objective was to study contact networks, and not to estimate population proportions from the sample.

Earlier we reported on a study in which we combined online respondent-driven detection with participatory surveillance, i.e., an Internet-based system that captures voluntarily submitted data on influenza-like-illness (ILI) symptoms from the general public [18]. We showed that such respondent-driven approach can be used to improve the detection of symptomatic cases [19]. In this paper we were interested in the contact networks of respondents and the association with self-reported disease. In particular, we aimed to determine correlations between participants linked by recruitment chains (i.e., who recruits whom) with respect to sociodemographic characteristics, vaccine-induced immunity and self-reported symptoms. In addition, we investigated the effect of spatial peer recruitment on these correlations. If recruitment of contact persons by participants is random, these statistical relationships reflect the underlying contact networks in the general population that are relevant for the transmission of respiratory pathogens.

## Methods

### Study design

Volunteers of two participatory surveillance panels were invited via the organizations’ electronic newsletters for an online and anonymous survey between November 2013 and May 2014. The first panel focused on ILI, operates in the Netherlands and Dutch speaking Flanders (Belgium), and had 16942 active volunteers. The second panel focused on pneumonia, operates only in the Netherlands, and had 1691 active volunteers. After completion of the questionnaire, participants were asked to recruit 4 contact persons (e.g., family members, friends, acquaintances) whom they had met face to face in the past 2 weeks. Invited contact persons were asked to do the same. Online peer recruitment was followed by means of unique codes that were automatically generated. Participants could invite contact persons via standard email, via a private message on Facebook, or by sharing a unique link (i.e., a Uniform Resource Locator that includes a personal code). A ‘seed’ indicates a volunteer from the surveillance panels who participated in our survey and a ‘recruit’ is a contact person recruited by a survey participant. By ‘waves’ we refer to consecutive subsamples, with seeds in wave 0, recruits invited by seeds in wave 1, and so forth. ‘Recruitment trees’ refers to chains of participants connected via recruitment. Invited contact persons could opt-out of the survey and provide reasons for not participating.

After completion of the questionnaire, participants were referred to a research website that displayed the latest results (e.g., anonymous network trees). Participants recruited via the first panel who completed the survey had the opportunity to join a raffle for 1 of 10 gift cards of €25. This incentive only slightly increased peer recruitment as was shown in Stein et al. [19]. For details on the software system and information on the 171 non-responders we also refer the reader to Stein et al. [19].

We obtained ethical approval from the Medical Ethical Committee of the University Medical Center Utrecht, The Netherlands (13-664/C). Informed consent was obtained before survey participation.

### Questionnaire

We defined ‘contact’ as touching a person (e.g., shaking hands or hugging) or talking to a person within a distance of about one arm’s length (duration of conversation did not matter). Participants were asked to report as precisely as possible the number of contact persons that they had during one full day (‘yesterday’) at four predefined locations, namely at home, at work or educational institute (school or university), at the house of family or friends or other acquaintances, and at other places (e.g., during sports, shopping or travelling, or in a restaurant or cafe). Participants were asked to specify the age group of the contact person (namely 0–11 years, 12–18 years, 19–60 years and older than 60 years); multiple contacts with the same person during the course of the day needed to be counted only once. ‘Degree’ denotes the total number of contact persons reported by a participant.

Participants were asked to report any symptoms (provided in a list) that they had experienced in the past 2 weeks. If any symptoms were reported, we asked additional disease related questions and whether they knew any contact persons with similar symptoms. Symptomatic participants were asked about the type of disease that they thought to have experienced (e.g., influenza or common cold); we further refer to this as self-reported influenza or common cold. We used the definition of the World Health Organization to define ILI that includes having fever (excluding questions on a body temperature of ≥ 38 C°) and cough with an onset within the last 10 days. Participants were also asked whether they had received an invitation to get an influenza vaccination and whether they had received influenza vaccination in the past 12 months. This information was used as a proxy for the possible immune status of participants. As earlier studies described clustered patterns of influenza vaccination uptake and sentiments concerning vaccination, we asked participants whether they believed that the influenza vaccine protects them against influenza [20, 21]. Lastly, for each participant we collected information on age, sex, educational level, household members and their age, four digit postal code, and work or study location. Parents could fill in the questionnaire for their child.

### Statistical analysis

First we assessed the main effects of covariates (age, sex, household size and ILI) on degree using a Poisson Inverse-Gaussian regression model (see also Additional file 1). This model is an alternative to a negative binomial model and has the potential for modelling highly dispersed count data due to the flexibility of the Inverse - Gaussian distribution [22, 23].

We investigated mixing patterns within our sample by analysing shortest paths between pairs of any two individuals that were one, two, or three or more link steps away from each other in the same recruitment tree [24]. Correlation coefficients with respect to the same measured variable were calculated for pairs of recruiter and recruit in consecutive waves. Pearson’s *r* was used for integer variables (age, degree and household size), phi coefficient (*r*_*φ*_) for binary variables (sex, vaccination status, symptoms) and Spearman rank-order (*r*_*rank*_) for ordinal variables (education, vaccination beliefs). These correlations provide both insight in recruitment patterns, as well as in clustering (i.e., contact persons of an individual with the same characteristic(s) are recruited or infected with a probability that is higher than expected if the distribution was random) of disease, vaccination status and sentiments.

We compared the sampled recruiter-recruit age matrix with the participant-contact age matrix collected in the Netherlands during POLYMOD (Van de Kassteele J, Van Eijkeren J, Wallinga J: Efficient estimation of age-specific social contact rates between men and women, in preparation) [9]. If we assume that POLYMOD data accurately reflects all contact persons of an individual, then by a comparison we can investigate to what extent recruitment links between two participants can be interpreted as a contact in the sense of our contact definition, at least with respect to age. Firstly, we used the two-sample Kolmogorov-Smirnov (KS) test to compare column wise for each participant’s age group the (integer) age distribution of recruits sampled in our study, with those of contact persons recorded in POLYMOD. Secondly, we used a homogeneous uniform association model (i.e., a model that assumes that all strata in two-way contingency tables have a common local odds ratio, OR) to test whether there is a statistical difference between both entire matrices [25–27].

To analyse the spatial spread of recruitment we converted the registered 4-digit postal codes into coordinates using geocoding and computed the distance between a recruiter and their recruit with the great-circle distance. We also computed the distance a participant commutes between home and the work or study location. We investigated the co-occurrence of a characteristic separately for recruiter-recruit pairs that had the same postal code, and between pairs that lived 1 to 10 km (km) and more than 10 km away from each other. The equality of correlation coefficients, calculated for integer variables, was tested using Fisher z-transformation [28]. The equality of odds ratios, calculated for binary variables, was tested using a log-linear model. Finally, we used a logistic regression model to estimate for individuals living in four different regions in the Netherlands the probability of recruiting a contact person at the work or study location (see also Additional file 1). Statistical analyses were performed in R (version 3.1.1).

## Results

### Description of sample

A total of 1560 individuals completed the survey at least once, of which 1105 seeds (wave 0) who were invited via the panels, and 455 recruits (waves 1 to 6) who were invited by participants. Neither participatory surveillance panel was representative of the general population in terms of basic demographic characteristics. However, through peer recruitment the sample representativeness slightly improved in terms of age and sex (see also Stein et al. [19]). Overall, 64.7 % of the participants were female, 55.5 % were aged between 50–69 years (mean age: 53.6; range: 3–97 years), 57.4 % obtained a bachelor degree or higher, 41.5 % had a two-person household and 41.9 % received an influenza vaccine in the past 12 months (Table 1). Less than half of all seeds (45.8 %) reported at least one symptom, while more than half of the recruits (on average 57.8 % in waves 1 to 6) reported symptoms. Of all participants, 8.3 % self-reported they had influenza of which 32.3 % had received the influenza vaccine, resulting in an OR of 0.64 [95 % confidence interval (CI) 0.42–0.95] for self-reported influenza by vaccinated individuals (compared to non-vaccinated).

**Table 1:** Sample characteristics overall and per recruitment wave

		Wave 0		Wave 1		Wave 2		Waves 3–6		Total	
		(n: 1105)		(n: 310)		(n: 93)		(n: 52)		(n: 1560)	
		n	%	n	%	n	%	n	%	n	%
Country	Netherlands	1018	92.1	295	95.2	86	92.5	52	100	1451	93.0
	Belgium	87	7.9	15^a^	4.8	7	7.5	0	0	109	7.0
Sex	Male	387	35.0	122	39.4	31	33.3	10	19.2	550	35.3
	Female	718	65.0	188	60.6	62	66.7	42	80.8	1010	64.7
Age^b^	0–39	139	12.5	91	29.3	26	28.0	13	25.0	268	17.2
	40–49	189	17.1	43	13.9	18	19.3	6	11.5	256	16.4
	50–64	496	44.9	106	34.2	32	34.4	22	42.3	656	42.1
	65+	281	25.5	70	22.6	17	18.3	11	21.2	379	24.3
Education	Bachelor or higher	651	58.9	166	53.5	56	60.2	23	44.2	896	57.4
	Lower than bachelor	144	41.1	29	46.5	37	39.8	29	55.8	664	42.6
Household^c^	1-person	280	25.3	78	25.2	22	23.7	10	19.2	390	25.0
	2-persons	478	43.3	110	35.5	38	40.9	22	42.3	648	41.5
	3-persons	145	13.1	35	11.3	6	6.4	6	11.5	192	12.3
	4 or more persons	202	18.3	87	28.0	27	29.0	14	26.9	330	21.2
Work or Study	Yes	775	70.1	228	73.5	73	78.5	41	78.8	1117	71.6
	No	330	29.9	82	26.5	20	21.5	11	21.2	443	28.4
Vaccinated^d^	Yes	516	46.7	104	33.5	19	20.4	15	28.8	654	41.9
	No	589	53.3	206	66.5	74	79.6	37	71.2	906	58.1
Symptoms	Yes	506	45.8	172	55.5	56	60.2	35	68.3	769	49.3
	No	599	54.2	138	44.5	37	39.8	17	32.7	791	50.7
Self-reported common cold	Yes	175	15.8	60	19.4	27	29.0	10	19.2	272	17.4
	No	930	84.2	250	80.6	66	71.0	42	80.8	1288	82.6
Self-reported influenza	Yes	96	8.7	24	7.7	7	7.5	3	5.8	130	8.3
	No	1009	91.3	286	92.3	86	92.5	49	94.2	1430	91.7
ILI	Yes	34	3.1	2	0.6	2	2.2	2	3.8	40	2.6
	No	1071	96.9	308	99.4	91	97.8	50	96.2	1520	97.4

^a^One participant lived in Germany

^b^One participant provided an invalid age

^c^Note: 48 participants who completed the survey did not provide information on their household size and were assumed to live alone

^d^Vaccinated against influenza in the past 12 months

### Reported contact persons

**Fig. 1:**
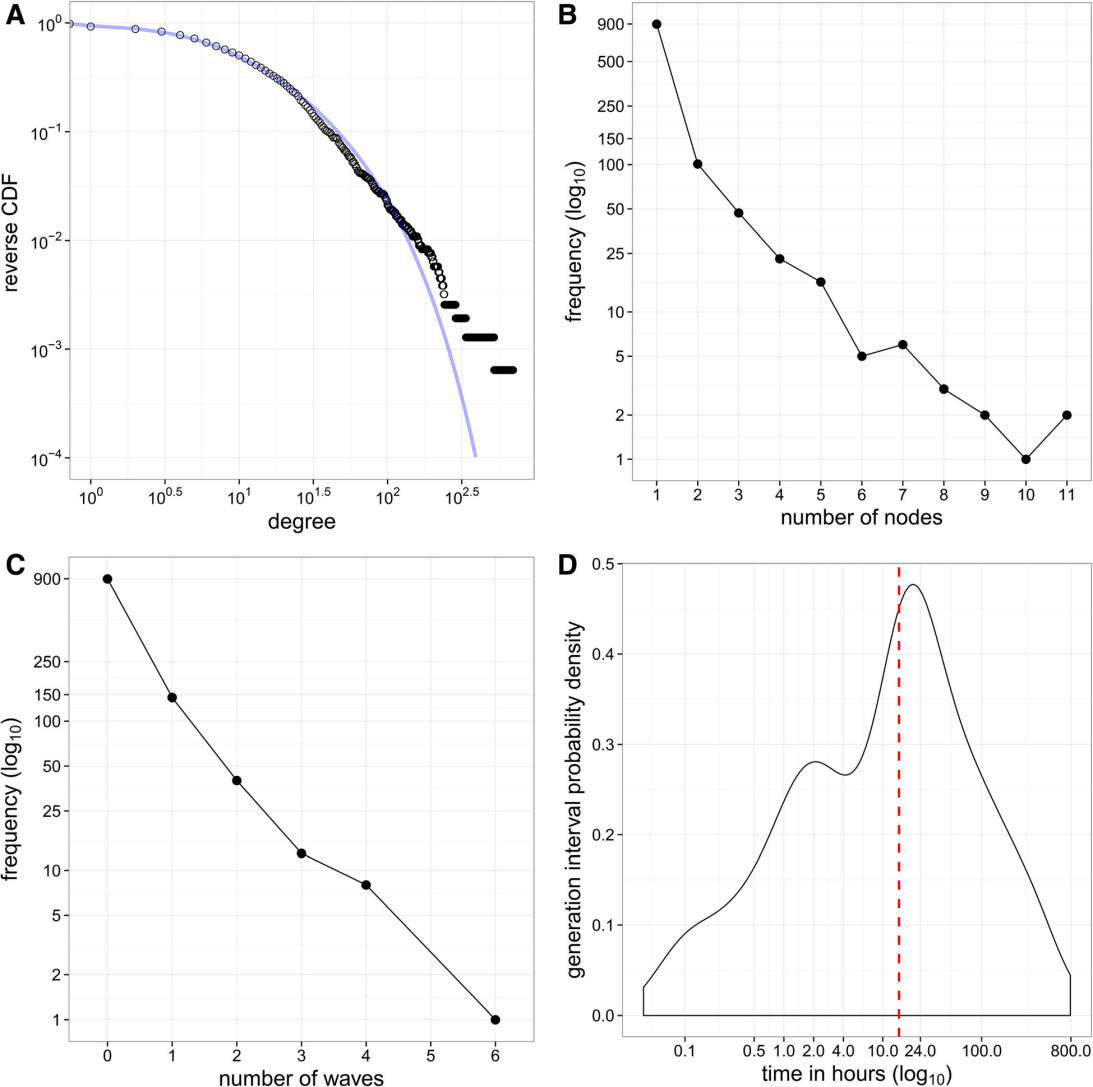
Reported contact persons and recruitment trees. **a** The empirical reversed cumulative distribution of degree (number of contact persons per participant) is indicated with black circles. The line is the fitted theoretical Poisson inverse-Gaussian distribution with mean *μ*: 19.6 (95 % CI 18.3–21.1) and dispersion parameter *λ*: 2.0 (95 % CI 1.8–2.1). **b** Number of participants (nodes) per recruitment tree. Most recruitment ‘trees’ only consisted of one participant (the seed), two trees consisted of 11 participants. **c** Number of waves that recruitment trees reached by peer recruitment, with seeds in wave 0. One recruitment tree reached 6 waves of recruits. **d** Recruitment generation interval. Red line indicates median generation interval

A total of 30591 contact persons were reported by 1531 participants, with a mean degree of 19.6 per participant (median: 11.0; standard deviation (SD): 35.3). Twenty-nine participants reported zero contact persons. Figure 1a displays the sampled degree distribution, which showed strong over-dispersion. A Poisson Inverse-Gaussian distribution with mean *μ* = 19.6 (95 % CI 18.3–21.1) and dispersion parameter *λ* = 2.0 (95 % CI 1.8–2.1) best fitted the empirical degree distribution. Analysis of degree with a multiple regression model showed a lower contact frequency for those aged ≥ 65 years compared to participants between 0 and 39 years old (Table 2). A larger household size was associated with a higher number of contact persons. Participants with ILI had less contact persons than persons without these symptoms. Such reduction in numbers of contacts has also been observed among ILI cases during the 2009 influenza epidemic and may be explained by people staying at home and avoiding social activities when ill [29]. Weekdays were associated with 33 %–84 % more contact persons than Sundays (see also Additional file 1 for the distribution of contact persons by days of the week), which is in accordance with results from other studies on contact patterns [9, 30].

**Table 2:** Number of reported contact persons per participant per day by different characteristics and relative number of contacts from the Poisson Inverse-Gaussian Regression model

Category	Covariate	Number of participants	Mean (standard deviation) of number of reported contacts	Relative number of reported contacts (95 % CI)^a^
Age of participant	0–39	268	20.98 (24.88)	1.00
	40–49	256	25.35 (37.24)	0.97 (0.80–1.17)
	50–64	656	19.94 (35.16)	0.93 (0.79–1.09)
	65+	379	14.19 (39.63)	0.69 (0.58–0.83)
Sex of participant	Female	1010	18.94 (30.78)	1.00
	Male	549	20.83 (42.41)	1.05 (0.94–1.18)
Household size	1	389	17.85 (29.49)	1.00
	2	648	15.73 (23.91)	1.02 (0.89–1.17)
	3	192	26.54 (58.17)	1.44 (1.20–1.73)
	4	218	24.93 (43.10)	1.55 (1.29–1.87)
	≥5	112	25.92 (37.37)	1.81 (1.43–2.29)
ILI	No	1519	19.93 (35.68)	1.00
	Yes	40	7.25 (9.70)	0.37 (0.25–0.53)
Days of the week	Sunday	224	16.68 (51.25)	1.00
	Monday	414	17.94 (32.15)	1.33 (1.12–1.59)
	Tuesday	249	24.27 (36.80)	1.84 (1.52–2.23)
	Wednesday	192	22.41 (31.73)	1.60 (1.30–1.96)
	Thursday	182	21.16 (28.29)	1.61 (1.31–1.99)
	Friday	117	18.76 (28.11)	1.42 (1.12–1.81)
	Saturday	181	16.65 (29.16)	1.27 (1.03–1.57)

^a^Dispersion parameter λ = 1.7 (95 % CI 1.4–2.1). The Poisson Inverse-Gaussian model is appropriate for modelling correlated counts with long sparse extended tails. The over-dispersion parameter in the model was significantly different from zero, indicating the necessity to use this model instead of a generalised Poisson model. Comparing AIC statistics, the Poisson Inverse-Gaussian model gave a better fit as opposed to a negative binomial model and a generalised Poisson model [22]

### Recruitment trees

Figure 1b shows the size of 1105 recruitment trees. Most recruitment trees consisted of only one node (i.e., seeds who did not recruit contact persons). There were 206 recruitment trees with at least two nodes (i.e., trees with at least two participants and one recruitment wave), and two of these trees consisted of 11 nodes each. One recruitment tree reached 6 waves of recruits. The majority of the recruits responded the same day they were invited by their recruiter, giving a median generation interval (i.e., the time between invitation by a recruiter and participation by his/her recruit) of 14.6 h (mean: 50.7; SD: 100.0) (Fig. 1d). Overall, the larger the proportion of women or individuals with a bachelor’s degree or higher in a recruitment tree, the larger the tree size was on average. Seed characteristics did not appear to influence the number of nodes in a recruitment tree (see also Additional file 1).

### Recruitment mixing patterns

Overall, we obtained 455 pairs between a recruiter and his/her recruit whereby both participants completed the survey. For an additional 33 pairs we solely obtained basic demographic information.

We observed assortative recruitment patterns by age (*r* = 0.36 [95 % CI 0.28–0.44]), education (*r*_*rank*_ = 0.31 [95 % CI 0.23–0.40]) and household size (*r* = 0.22 [95 % CI 0.13–0.30]), indicating that participants tend to recruit contact persons similar to themselves (Table 3). Recruitment was random (i.e., not assortative, nor disassortative) by sex (*r*_*φ*_ = 0.07 [95 % CI −0.02–0.16]) and degree (*r* = 0.07 [95 % CI −0.03–0.16]).

**Table 3:** Homophily in network components for different link steps

	Variables (type of correlation coefficient)	1 link step^a^	*p* value	2 link steps^a^	*p* value	3-6 link steps (lumped together)^a^	*p* value
Type of contact network	Age (*r*)	0.36 [0.28–0.44]	<0.001 (df: 486)	0.13 [−0.03−0.28]	0.109 (df: 156)	0.23 [−0.01−0.43]	0.058 (df: 70)
	Sex (*r*_*φ*_)	0.07 [−0.02–0.16]	0.107 (df: 486)	0.25 [0.09–0.39]	0.002 (df: 156)	0.17 [−0.07−0.38]	0.167 (df: 70)
	Education (*r*_*rank*_)	0.31 [0.23–0.40]	<0.001 (n: 488)	0.08 [−0.08–0.24]	0.293 (n: 158)	−0.01 [−0.25−0.21]	0.951 (n: 72)
	Household size (*r*)	0.22 [0.13–0.30]	<0.001 (df: 486)	0.18 [0.02–0.33]	0.025 (df: 156)	0.03 [−0.20−0.26]	0.785 (df: 70)
	Degree LOG (*r*)	0.07 [−0.03–0.16]	0.153 (df: 468)	−0.02 [−0.18–0.14]	0.808 (df: 149)	−0.03 [−0.26−0.21]	0.838 (df: 67)
Clustering of vaccination and disease	Vaccinated (*r*_*φ*_)	0.23 [0.14–0.32]	<0.001 (df: 453)	0.02 [−0.14–0.18]	0.817 (df: 143)	0.07 [−0.17−0.30]	0.567 (df: 67)
	Belief vaccination protects (*r*_*rank*_)	0.26 [0.18–0.35]	<0.001 (n: 455)	0.02 [−0.14–0.18]	0.812 (n: 145)	0.11 [−0.13−0.32]	0.387 (n: 69)
	One or more symptoms (*r*_*φ*_)	0.11 [0.02–0.20]	0.018 (df: 453)	0.11 [−0.05–0.27]	0.179 (df: 143)	0.15 [−0.09−0.37]	0.231 (df: 67)
	Self-reported common cold (*r*_*φ*_)	0.04 [−0.06–0.13]	0.455 (df: 453)	−0.08 [−0.24–0.08]	0.333 (df: 143)	−0.11 [−0.33−0.14]	0.389 (df: 67)
	Self-reported influenza (*r*_*φ*_)	0.26 [0.17–0.34]	<0.001 (df: 453)	0.03 [−0.13–0.20]	0.691 (df: 143)	−0.04 [−0.27−0.20]	0.764 (df: 67)

^a^Coefficients and 95 % confidence intervals are shown

Pairs showed frequently a similar influenza vaccination status (*r*_*φ*_ = 0.23 [95 % CI 0.14–0.32]) and the same beliefs on vaccine effectiveness (*r*_*rank*_ = 0.26 [95 % CI 0.18–0.35]). To a lesser extent, we observed assortative recruitment by self-reported symptoms (*r*_*φ*_ = 0.11 [95 % CI 0.02–0.20]). There were 150 (33.0 %) pairs where both individuals reported at least one symptom compared to 104 (22.9 %) where both did not report any symptoms.

The assortative correlations by age persisted between any two participants that were two or more link steps away from each other in the same network chain, indicating that the survey mainly spread among individuals of similar age. Having one or more symptoms also seemed to cluster within the same recruitment trees.

### Comparison with POLYMOD

**Fig. 2:**
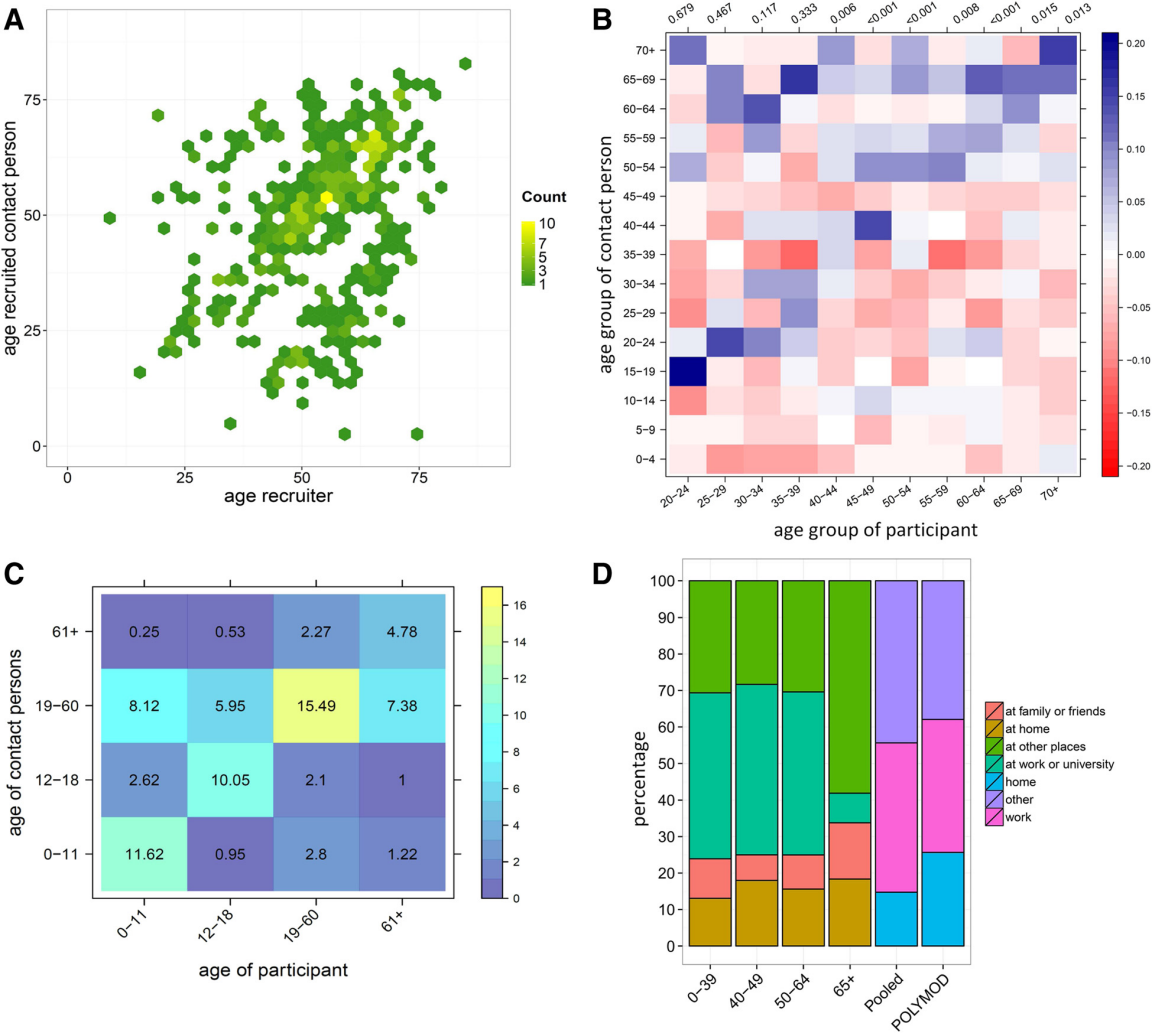
Recruitment and contact persons by age. **a** Recruitment patterns by age (n_pairs_: 488). **b** Difference between recruitment matrix and contact matrix by age of Dutch POLYMOD. Colours and scale indicate for each cell the proportional difference between both matrices, for the particular participant’s age group and his/her contact person’s age group (note: recruitment matrix minus POLYMOD matrix). For each participant’s age group, integer counts of contact persons were compared with POLYMOD using a two-sample KS test, the p values are shown above each column. **c** Contact persons reported in questionnaire by participants, values indicate the average number of contact persons in an age group recorded per day by participants. **d** Contact location by age groups and pooled for comparison with POLYMOD. The first four columns show the locations as displayed in the questionnaire. For comparison with POLYMOD, the sample was weighted for the size of POLYMOD age groups (weights are displayed in Additional file 1), and the category “at the home of family and friends” was combined with “other”. POLYMOD was regrouped as “home”, “work” (at work and at school combined) and “other” (leisure, travel and other combined), frequency of contact with the same person was ignored and for contact at multiple locations only the first entry was counted (equivalent to our questionnaire)

Figure 2a shows the recruiter-recruit matrix by age that visualizes the strong tendency of participants to recruit contact persons of similar age. This pattern is most pronounced in those aged 50–65 years. We observed two sub-diagonals that represent recruitment across generations. A column wise comparison with the contact mixing matrix by age of POLYMOD showed comparable distributions for participants aged between 20–39 years (Fig. 2b). This suggests that recruitment links might be representative for the contact persons recruiters encounter in daily life, at least with respect to age. However, the number of recruitments by participants in this age group was likely insufficient for a proper comparison of samples. A statistical comparison of the entire two matrices showed a significant difference (*p* < 0.001).

Overall, the strong assortative recruitment by age resulted in higher sample proportions of recruits of similar ages, while pairs of individuals with different ages were underrepresented compared to POLYMOD. The average numbers of contact persons by age reported in the questionnaire by participants were consistent with the assortative recruitment patterns. This was most apparent for participants aged between 19–60 years who reported mainly contact with persons of the same age group (Fig. 2c).

Participants below the age of 65 years mostly reported contacts at work or university, while those aged ≥ 65 years reported mostly contacts at other places. The number of persons contacted at different locations was similar in POLYMOD, although participants in our sample reported slightly less contact persons at home (Fig. 2d).

In the Additional file 1 we displayed the mixing matrices by age of our sample and of POLYMOD separately, as well as the absolute number of self-reported symptoms and a visualisation of the mixing patterns by degree.

### Spatial recruitment

**Fig. 3:**
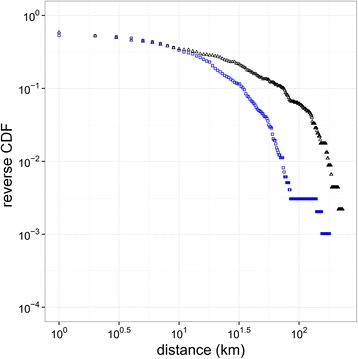
Distribution of recruitment and commuting distances. Black triangles indicate distances between recruiters and their recruits, with median 2.8 km (mean: 20.7; SD: 38.3). Blue squares indicate distances participants commute to work, with median: 3.4 km (mean: 11.0; SD: 18.1)

The median geographical distance between a recruiter and recruit was 3.0 km (mean: 21.0; SD: 38.5) (Fig. 3). There were 180 recruits with the same postal code as their recruiter, which suggests recruitment of nearby residents including household members. Seeds and their recruits lived on average further away from each other than pairs of participants in consecutive waves. The mean distance decreased from 22.4 km (SD: 40.1) between participants in waves 0 and 1, to 14.6 km (SD: 27.1) between participants in waves 2 and 3.

**Fig. 4:**
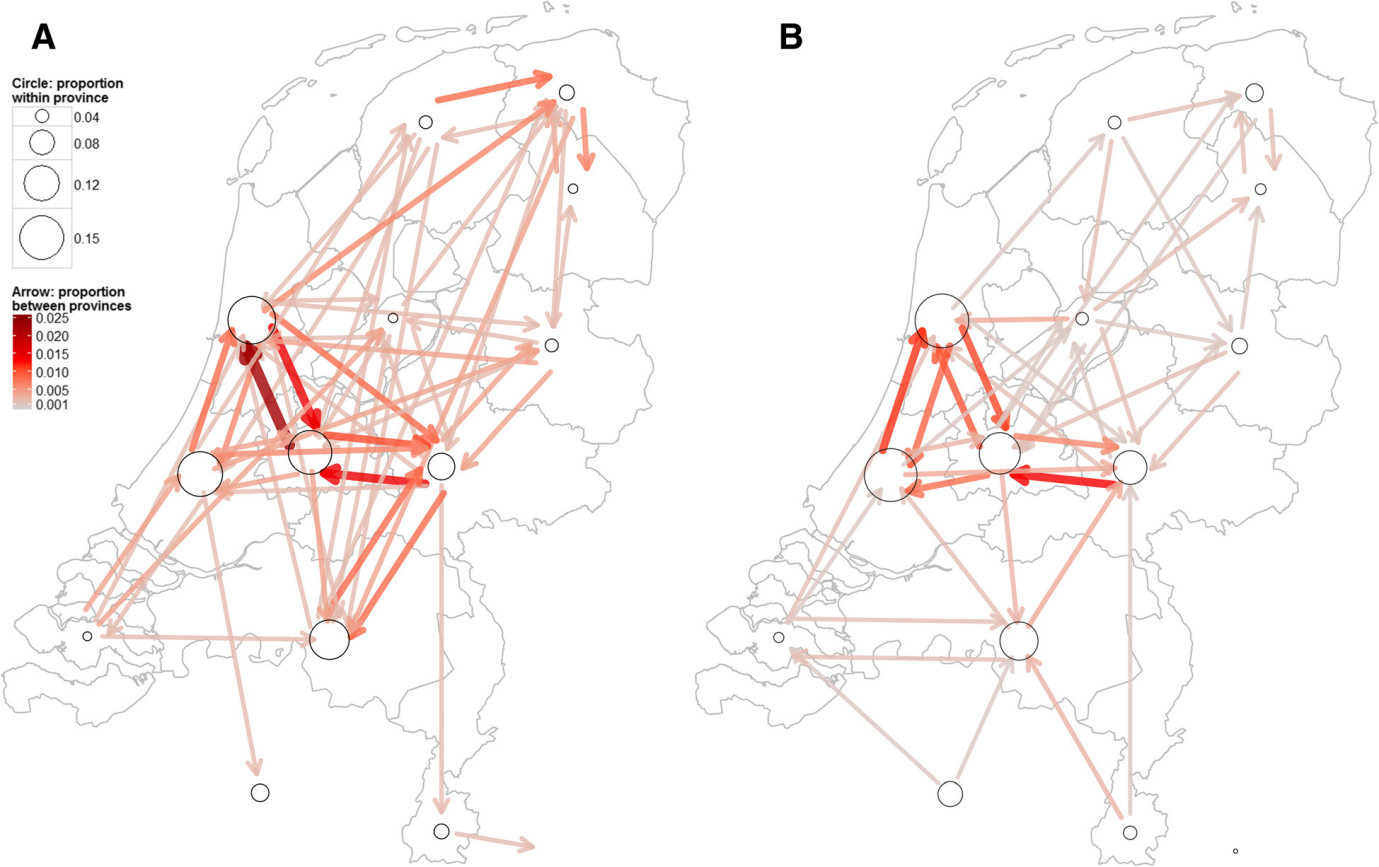
Spatial recruitment and commuting network structure. **a** Peer recruitment within The Netherlands and (between) Belgium. Arrows indicate recruitment between provinces and circles recruitment within a province. **b** Commuting network: directions that participants daily commute to work or study. Arrows indicate commuting across provinces, and circles commuting within a province. Sizes of arrows and circles are weighted for the total number of recruitments/commuters, with darker colours/larger circles indicating higher proportions. The maps were created with a shapefile (.shp file) that was extracted from GADM, an online geographic database of global administrative areas that is freely available for academic and other non-commercial use [45]

Of all recruitments, 76.4 % took place within the same Dutch province (i.e., the Netherlands counts 12 provinces that represent the administrative layers between the national government and the local municipalities) or within Belgium (included as one ‘province’), which corresponds to the 87.7 % of all participants that work or study within their province of residence (Fig. 4). The estimated probabilities of recruiting a contact person in the municipality where the recruiter both lived *and* worked varied between 0.56–0.77 (see also Additional file 1).

The distance between a recruiter and recruit determined the type of contact networks being sampled. Recruitment of persons with same postal code was stronger assortative by age, education, household size, degree, vaccination status and vaccination beliefs, and strongly disassortative by sex, compared to recruitment of persons who lived 1 km or further away. These patterns may reflect recruitment of individuals within the same household, such as partners. Participants were more likely to recruit persons of the same sex who lived 1 km or further away. Recruitment was strongly assortative by vaccination beliefs for pairs living >10 km away from each other, and by one or more symptoms and self-reported influenza for pairs living 1 to 10 km away from each other (Table 4).

**Table 4:** Effect of geographical distance on recruiter-recruit^a^ relationship

Variable	correlation/odds ratio	Same postal code^b^	*p* value	1 to 10 km^b^	*p* value	>10 km^b^	*p* value	Overall test
Age	*r*	0.50 [0.39–0.61]	<0.001 (df: 177)	0.40 [0.25–0.53]	<0.001 (df: 144)	0.21 [0.06–0.35]	0.008 (df: 160)	0.008
Education	*r*_*rank*_	0.33 [0.19–0.47]	<0.001 (n: 179)	0.26 [0.09–0.41]	0.001 (n: 146)	0.32 [0.15–0.47]	<0.001 (n: 162)	0.770
Household size	*r*	0.40 [0.26–0.51]	<0.001 (df: 177)	0.08 [−0.09–0.24]	0.363 (df: 144)	0.14 [−0.01–0.29]	0.067 (df: 160)	0.004
Degree LOG	*r*	0.16 [0.01–0.30]	0.034 (df: 173)	−0.02 [−0.18–0.15]	0.855 (df: 136)	0.04 [−0.11–0.20]	0.583 (df: 154)	0.264
Belief vaccination protects	*r*_*rank*_	0.19 [0.04–0.35]	0.012 (n: 169)	0.17 [−0.00–0.33]	0.056 (n: 131)	0.41 [0.27–0.55]	<0.001 (n: 154)	0.041
Sex	OR	0.35 [0.14–0.79]	0.006 (n: 179)	4.86 [2.13–11.39]	<0.001 (n: 146)	1.91 [0.93–3.93]	0.054 (n: 162)	<0.001
Vaccinated	OR	4.94 [2.30–11.07]	<0.001 (n: 169)	3.54 [1.50–8.67]	0.001 (n: 131)	1.36 [0.66–2.81]	0.366 (n: 154)	0.025
One or more symptoms	OR	1.09 [0.57–2.11]	0.771 (n: 169)	3.03 [1.39–6.80]	0.002 (n: 131)	1.36 [0.68–2.72]	0.349 (n: 154)	0.093
Self-reported common cold	OR	1.27 [0.47–3.23]	0.585 (n: 169)	1.31 [0.33–4.35]	0.635 (n: 131)	1.10 [0.25–3.79]	0.874 (n: 154)	0.974
Self-reported influenza	OR	8.01 [1.98–31.38]	<0.001 (n: 169)	9.32 [1.22–59.64]	0.001 (n: 131)	4.90 [0.73–25.05]	0.052^c^ (n: 154)	0.814

^a^Number of pairs with same postal code (n: 180 pairs), with same Internet Protocol (IP) address (n: 86), and number of pairs with both same postal code and same IP address (n: 72)

^b^Correlation coefficients/odds ratios with 95 % confidence intervals are shown

^c^Fisher’s exact test was used for contingency tables containing small values (*n* < 10)

## Discussion

In this study we explored social contact networks arising from a respondent-driven survey conducted in the Netherlands and parts of Belgium during the winter season 2013–2014. We have shown that an online respondent-driven method in combination with participatory surveillance can be used to (i) study contact networks relevant for the spread of infectious diseases that transmit via close contact between individuals, (ii) detect clustering of these diseases in a contact network, and (iii) reach within short time and with large spatial coverage a diverse group of individuals in the general population. Furthermore, we found that the spatial distribution of recruitment influences the type of contact networks being sampled.

We analysed a large number of recruiter-recruit pairs and of individuals with different ages and backgrounds. This enabled us to investigate the distribution of numbers of contact persons and to quantify the strength of network ties that allow the transmission of diseases that spread via close contact or airborne droplets. Such information can inform mathematical models of infectious disease epidemics [31–34]. Symptomatic participants showed a tendency to recruit other symptomatic participants, at least for one or more symptoms and self-reported influenza. This observation lends some support to our hypothesis that via respondent-driven recruitment we reached contact persons whom a participant may infect or by whom a participant may get infected in case of an infectious disease outbreak. The self-reported symptom data by pairs of participants provides an indication on disease clustering in contact networks. Such information can be quickly obtained with online respondent-driven detection as the recruitment generation interval was less than one day.

We also observed clustering of the same influenza vaccination status and reported sentiments about vaccination in recruitment trees. Such clustering of similar health behaviour has been described before and provides an indication of clustering of vaccine-induced immunity in a population [20, 21]. Clustering of negative vaccination statuses or sentiments about vaccination leads to clusters of unprotected individuals that increase the likelihood of disease outbreaks [21]. Such information could be used to design intervention messages for vulnerable populations.

Compared to a paper-based approach [17], online peer recruitment was spatially wider dispersed and covered a larger geographical area. A stratification on distance of the relationships between recruiter-recruit pairs showed differences in the type of recruited contact persons. There may be several explanations why a participant invited certain contact persons [35]. For example, symptomatic participants may have been biased towards inviting symptomatic contact persons who lived further away than contact persons whom they more frequently meet. A proper assessment would require to investigate the ‘pool of contact persons’ from which a recruiter can choose, and which contact persons were invited but did not join the survey. Furthermore, identifying different types of relations (e.g., family members, friends or colleagues) by asking recruits about their recruiter would allow further clarification of the observed correlations. Such information can only be collected with a non-anonymous survey design, which would also make it possible to measure transitivity, i.e., the extent to which contact persons of a participant are also contact persons of each other [36]. This network property is known to reduce the rate at which an infection can spread through a network [36–38].

The ‘who recruited whom’ matrix stratified by age showed qualitatively similar structures as the contact matrix by age reported in POLYMOD [9]. In addition, proportions of contact persons at different locations were similar to POLYMOD and the regression analysis showed similar covariates such as age, household size and days of week to affect degree. This suggests for online recruitment that invited contact persons are in general representative for the contact persons daily encountered by participants and that respondent-driven detection can indeed provide accurate information on the underlying contact network. However, despite the fact that recruitment criteria were set the same for all participants, regardless of whether they reported symptoms, we cannot preclude a bias in how participants chose from their contact persons. The age matrices were statistically not comparable. There may be several explanations for this statistical discrepancy, such as a difference in the age distributions of the samples and the fact that POLYMOD participants were able to report an unrestricted number of contact persons, while our survey participants could only invite a maximum of four contact persons.

This study has limitations. By using participatory surveillance panels for recruitment of seeds, we reached a diverse group of individuals within a short period of time. However, the volunteers in these panels are not representative for the general population; some groups like women and highly educated persons are overrepresented [19]. Such overrepresentations are common in participatory surveillance systems [18]. We did reach all age groups, but due to strong assortative peer recruitment certain age classes were represented more in the sample and the young age classes were reached less with our survey, therefore limiting the generalisability of our results to the young age groups.

To reduce the participation burden and stimulate recruitment at the end of the questionnaire, we applied an aggregated contact diary design, i.e., a participant did not need to report on each contact separately. The mean number of contact persons per participant was therefore likely higher than in previous studies [9, 39]. More importantly, we did not collect information on contact intensity and duration. The probability of transmission between individuals requires different levels of contact for different infectious diseases, e.g., influenza and measles require only spatial proximity between individuals to transmit, while Ebola is believed to require physical contact to cause infection [7, 14]. Note that the survey did not include questions on other potentially important transmission routes, such as exposure not involving physical contact or conversation (e.g., sneezing passenger in public transport) or indirect fomite transmission from shared contaminated objects [7]. Earlier studies explicitly linked contact intensity and duration with infection risk and showed their importance for understanding transmission dynamics [40, 41]. Contact duration also influences the likelihood that a certain contact is reported, e.g., contacts of long duration are substantially more likely to get reported than contacts of short duration [42, 43]. It is possible to derive these contact metrics from earlier studies, but not to exclude the effect of heterogeneities in motivation or recall capabilities on reported numbers of contacts, e.g., between male and female participants [42].

In a future survey volunteers of participatory surveillance panels could be selected according to specific characteristics to obtain seeds that are in some sense representative for the general population. Furthermore, it may be useful to conduct a similar study in other countries where comparable participatory surveillance systems are in place, such as the United Kingdom, Italy and France, to allow for a country comparison [44].

## Conclusions

In this study we used online respondent-driven detection to study the distribution of the number of contact persons and mixing patterns within contact networks. The observed contact patterns are relevant for the transmission of respiratory pathogens that spread via close contact between individuals. We found that the spatial distribution of recruitment influenced the type of contact networks being sampled. Even though complex mechanisms influence peer recruitment, the observed statistical relationships reflected the observed contact network patterns in the general population. This provides useful and innovative input for predictive epidemic models relying on network information.

## Additional file

Additional file 1:
**Tracking social contact networks with online respondent-driven detection: who recruits whom?** This file contains supporting information for the results presented in the manuscript “Tracking social contact networks with online respondent-driven detection: who recruits whom?”. (PDF 2053 kb)

## Abbreviations

CI: Confidence interval
ILI: Influenza-like-illness
IP: Internet Protocol
km: kilometres
KS: Kolmogorov-Smirnov
OR: Odds ratio
SD: Standard deviation
