# Supplementary material for: Tracking social contact networks with online respondent-driven detection: who recruits whom?
Source: BMC Infect Dis. 2015 Nov 14;15:522. doi: 10.1186/s12879-015-1250-z (PMC4647802; doi:10.1186/s12879-015-1250-z)
Supplement: Additional file 1: — Tracking social contact networks with online respondent-driven detection: who recruits whom? This file contains supporting information for the results presented in the manuscript “Tracking social contact networks with online respondent-driven detection: who recruits whom?”. (PDF 2053 kb) [file 12879_2015_1250_MOESM1_ESM.pdf]

ADDITIONAL FILE

Accompanying to the manuscript entitled "Tracking social contact networks with online respondent-driven detection: who recruits whom?"

Mart L. Stein[1,2*], Peter G.M. van der Heijden[3,4], Vincent Buskens[5], Jim E. van Steenbergen[2,6], Linus Bengtsson[7,8], Carl E. Koppeschaar[9], Anna Thorson[7], Mirjam E.E. Kretzschmar[1,2]

1. Julius Center for Health Sciences and Primary Care, University Medical Center Utrecht, the Netherlands
2. Centre for Infectious Disease Control, National Institute for Public Health and the Environment, the Netherlands
3. Department of Methodology and Statistics, Faculty of Social and Behavioural Sciences, University Utrecht, the Netherlands
4. Southampton Statistical Sciences Research Institute, University of Southampton, United Kingdom
5. Department of Sociology, Faculty of Social and Behavioural Sciences, University Utrecht, the Netherlands
6. Centre of Infectious Diseases, Leiden University Medical Centre, the Netherlands
7. Department of Public Health Sciences-Global Health, Karolinska Institutet, Sweden
8. Flowminder Foundation, Sweden
9. Science in Action BV, the Netherlands

M.L. Stein, et al.
m.l.stein-2@umcutrecht.nl

Table of Contents

This file contains supporting information for the results presented in the manuscript "Tracking social contact networks with online respondent-driven detection: who recruits whom?". The supportive information is presented in the order as it is discussed in the main manuscript.

In chapter 1 we explained in detail how numbers of contacts and the effects of covariates were analysed. In chapter 2 we investigated the influence of participants' characteristics on the size of a recruitment tree. In chapter 3 we displayed the mixing matrices by age of our sample and of the Dutch POLYMOD separately. Here we also provided the absolute number of self-reported symptoms and a visualisation of the mixing patterns by degree. In chapter 4 we analysed the distance between recruiters and their recruits, and quantified the extent to which a recruiter who lives in a certain region in the Netherlands invited contact persons that live in the same municipality as the recruiter is working and/or studying.

1. Numbers of contact persons and the effect of covariates 2
2. Descriptive analysis of recruitment trees 5
3. Recruitment mixing patterns 8
    3.1 Mixing by age and comparison with POLYMOD 8
    3.2 Mixing by degree 10
    3.3 Mixing by self-reported symptoms 11
4. Distance between recruiter-recruit pairs 12
    4.1 A logistic regression analysis 14
References 17

1. Numbers of contact persons and the effect of covariates

In the questionnaire participants were asked for number of contact persons during one full day ('yesterday'), this number was defined to be a participant's 'degree'. First, we looked at the distribution of degree, stratified by days of week and the locations that were predefined in the questionnaire (Figure A1). For at home and at other places the distributions of degree were fairly similar. During weekdays participants reported more contact persons at work or university, then during the weekend. There were no large differences in the total degree distributions (see 'at all locations') between weekdays and weekends.

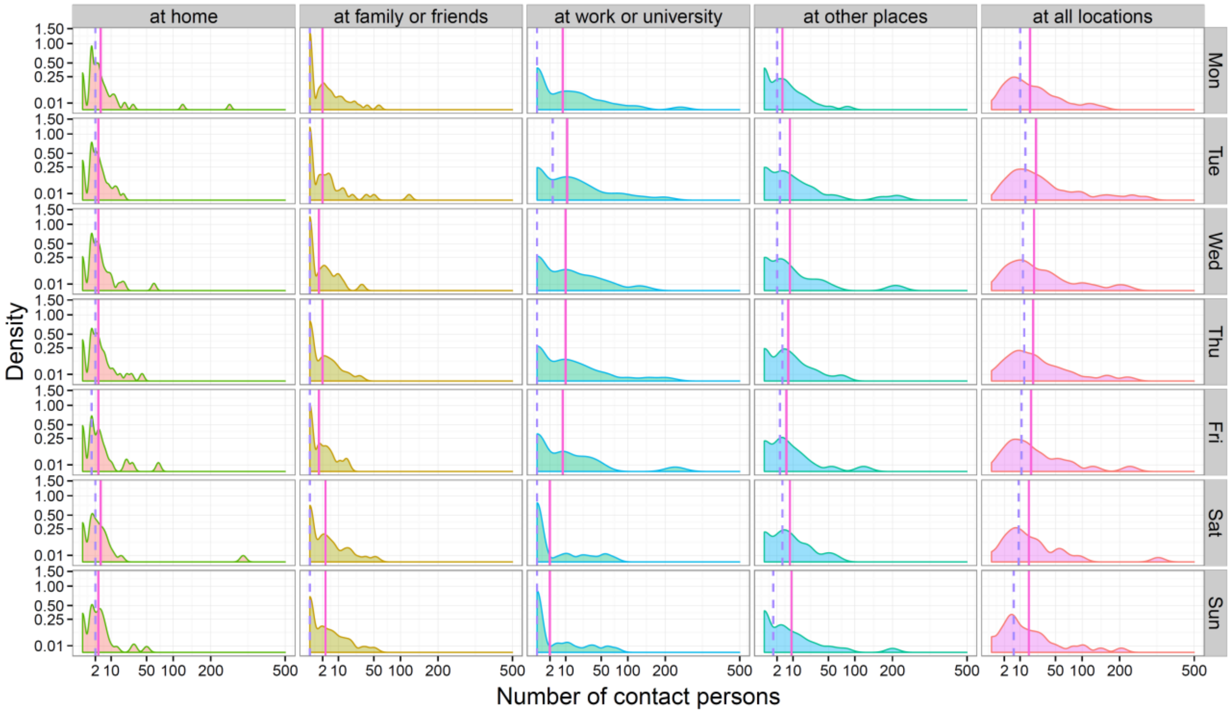

Figure A1. Contact persons stratified by days of the week and location of contact. In each distribution the mean (solid line) and median (dotted line) is displayed. Participants with more than 500 contact persons are not displayed.

We investigated which covariates influence degree using a regression model. Firstly, we investigated which theoretical distribution best fitted the empirical distribution using the R package 'GAMLSS'. The degree distribution showed strong over-dispersion, with a mean degree of 19.6 per participant (median: 11.0; SD: 35.3). Table A1 displays the parameter estimates and AIC's of the various fitted distributions. Note that the power-law was fitted with the GAMLSS function "PARETO2". Cumulative distributions with a power-law form are sometimes said to follow a Pareto distribution (or Zipf 's law) [1]. Figure A2 displays the various distributions in a reverse cumulative probability distribution plot (Log_{10} transformed).

Table A1. Theoretical distributions fitted to empirical degree distribution.

	parameter(s)	AIC
Pareto 2 (α; x_m)[a]	49.46; 0.28	12179
Log normal (μ; σ)[a]	11.67; 1.03	12184
Poisson Inverse Gaussian (μ; *λ*)	19.62; 1.96	12207
Negative binomial (μ; k)	19.61; 1.12	12475
Geometric (discrete) exponential (*p*)	19.61	12485
Poisson (*λ*)	19.61	48667

[a] Continuous distributions fitted to a discrete distribution.

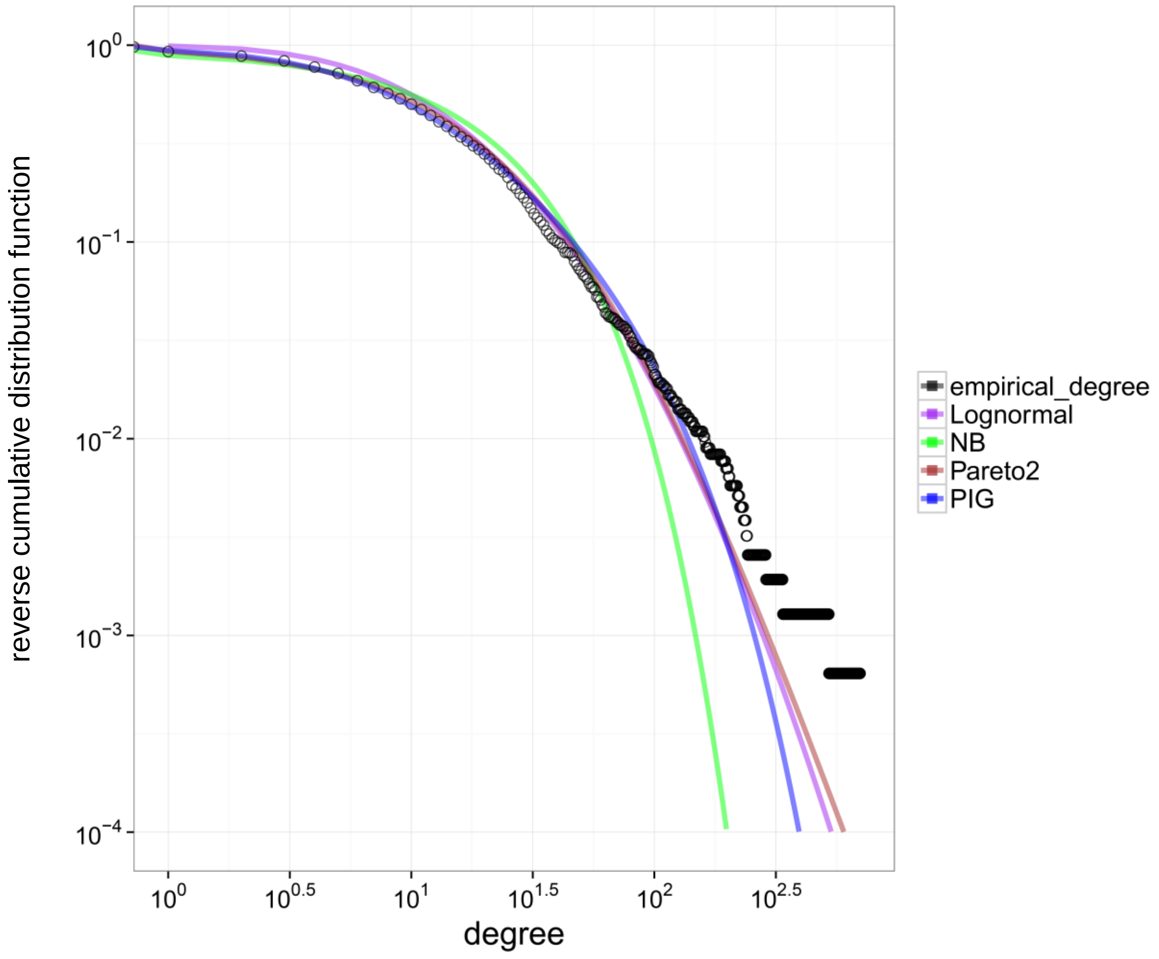

Figure A2. Fitted theoretical distributions to empirical degree distribution. The empirical degree distribution (number of reported contact persons) is displayed with black circles. The figure displays four theoretical distributions that were fitted to the empirical degree distribution, namely: the Poisson-inverse Gaussian distribution (PIG, discrete distribution), the Negative Binomial distribution (NB, discrete distribution), the Pareto 2 distribution (continuous distribution) and the Log-normal distribution (continuous distribution).

Based on the AIC's, the continuous distributions Log-normal and Power-law best fitted the empirical degree distribution [2]. However, these are continuous distributions fitted to a discrete distribution. Therefore, we chose the first best fitted discrete distribution: the Poisson-inverse Gaussian (PIG). The PIG distribution, an alternative to negative binomial, has the potential for modelling highly dispersed count data due to the flexibility of Inverse Gaussian distribution [3, 4]. We applied the PIG distribution in the regression analysis.

We used a PIG regression model to investigate the effect of the following covariates on degree: age, sex, household size and ILI, and days of the week. The reference categories were the 0–39 age group, females, one-person households, no self-reported ILI, and Sunday. Table A2 shows the output of the regression model. IRR stands for incidence rate ratio that are standard provided when conducting a PIG regression analysis.

Table A2. Output Poisson Inverse-Gaussian regression model

	IRR[a]	SE	t value	Pr(>|z|)	2.5%	97.5%
Intercept	13.055	0.109	23.527	0.000	10.540	16.171
40-49	0.969	0.094	-0.338	0.735	0.805	1.166
50-64	0.928	0.081	-0.918	0.359	0.791	1.089
65+	0.692	0.093	-3.985	0.000	0.577	0.829
male	1.053	0.058	0.887	0.375	0.940	1.180
household size:2	1.019	0.070	0.263	0.792	0.887	1.170
household size:3	1.441	0.094	3.896	0.000	1.199	1.732
household size:4	1.552	0.094	4.655	0.000	1.290	1.867
household size:5 or more	1.809	0.121	4.888	0.000	1.426	2.294
ILI	0.367	0.188	-5.320	0.000	0.254	0.531
Monday	1.334	0.089	3.237	0.001	1.120	1.588
Tuesday	1.837	0.098	6.192	0.000	1.515	2.226
Wednesday	1.597	0.105	4.469	0.000	1.301	1.961
Thursday	1.615	0.107	4.470	0.000	1.309	1.993
Friday	1.423	0.122	2.897	0.004	1.121	1.806
Saturday	1.269	0.109	2.197	0.028	1.026	1.570

[a] IRR: incidence rate ratio. Number of observations: 1559, df: 17 AIC: 12050.81, Global deviance: 12016.81

2. Descriptive analysis of recruitment trees

We conducted a descriptive analysis to investigate which characteristics of individuals in a recruitment tree influence the total size of a tree. Firstly, we plotted the number of nodes, i.e., participants who completed the questionnaire, stratified by characteristics of seeds (Figure A3).

The characteristics of seeds did not appear to influence the number of nodes in a recruitment tree. Figure A3-B did show a slight increase in tree size for a larger proportion of trees with a female seed, e.g., of all trees with a node size of 4 more than 75% had a female seed. This effect of female seeds was not shown for trees with a size of 5 or more nodes, which is probably due to the lower number of trees with those sizes.

In Figure A4 we investigated the relationship between tree size and the composition of the entire recruitment tree. Overall, the larger the proportion of women or individuals with a bachelor's degree or higher in a recruitment tree, the larger the tree size was on average (see Figures A4-A to C). The average age in a recruitment tree did not appear to influence the number of nodes in a recruitment tree (Figure A4-D).

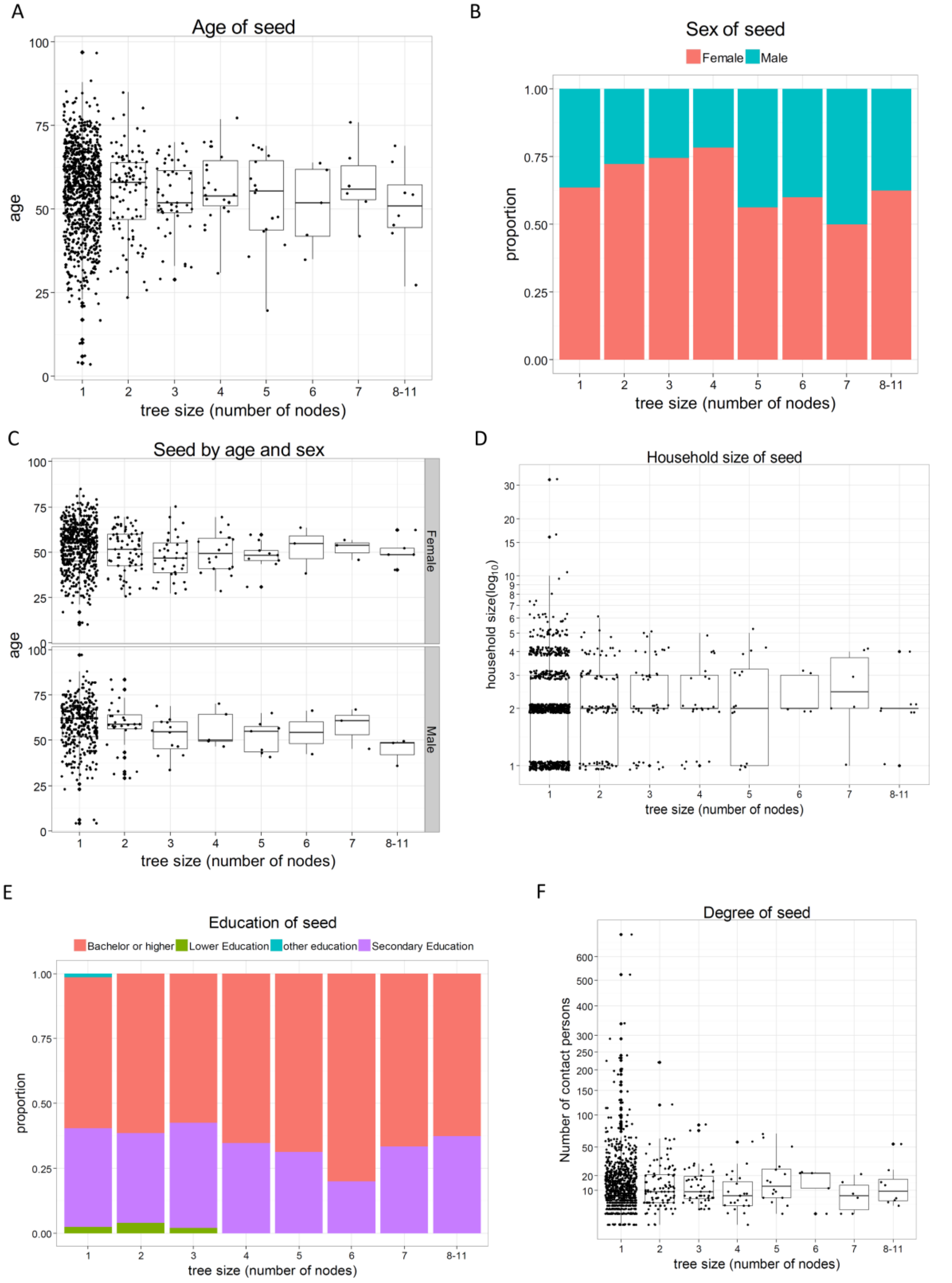

Figure A3. Relation between tree size and characteristics of *seeds*. With **(A)** age of seeds, (**B**) sex of seeds, (**C**) age and sex, (**D**) household size, (**E**) educational level of seeds, (**F**) number of contacts of seeds (degree).

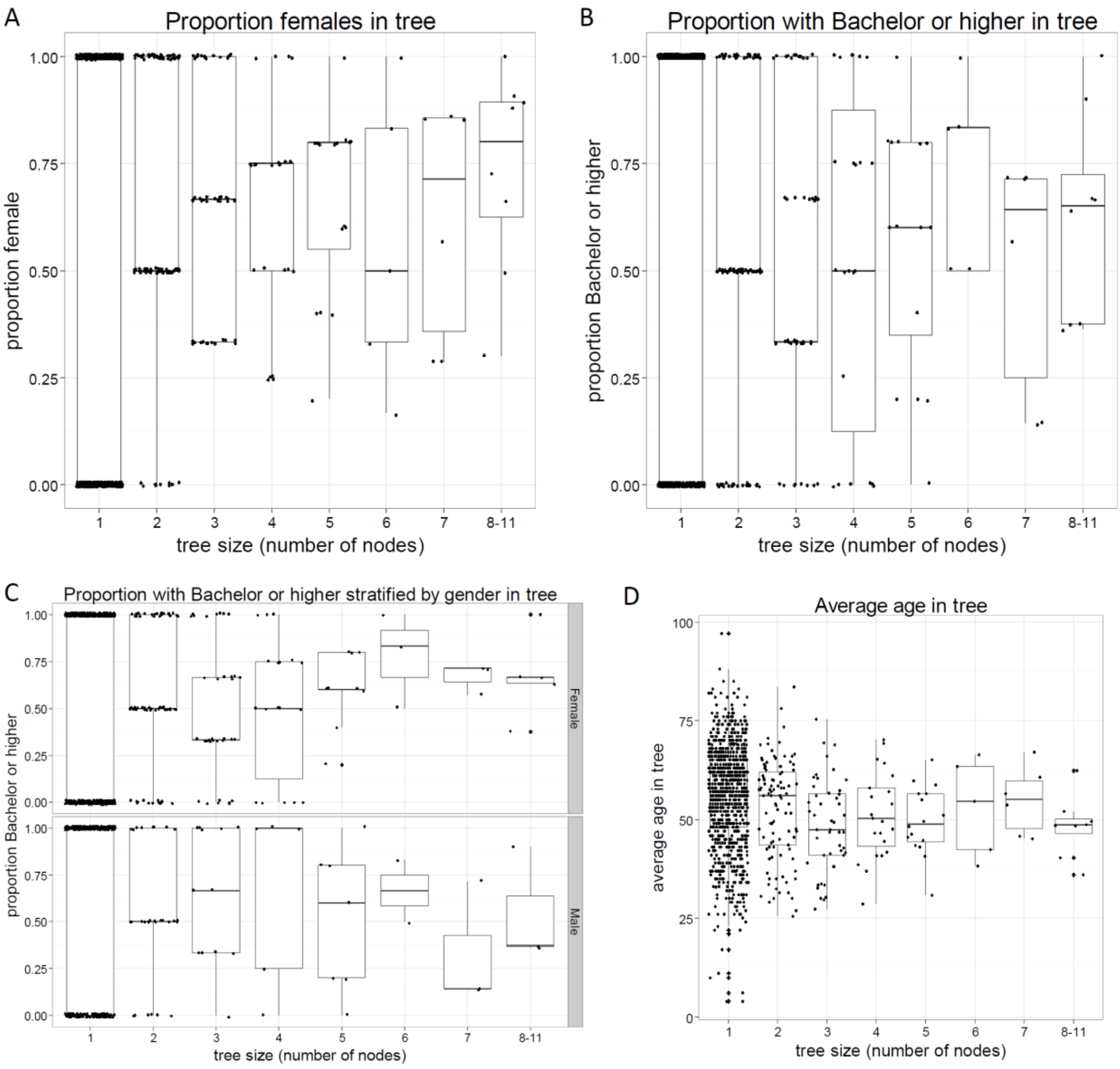

Figure A4. Relation between tree size and composition of trees. With (**A**) proportion of females in a tree, (**B**) proportion of individuals with a bachelor's degree or higher, (**C**) tree size stratified by number of individuals in a tree with a bachelor's degree or higher and sex, (**D**) the average age of individuals in one tree.

3. Recruitment mixing patterns

3.1 Mixing by age and comparison with POLYMOD

We compared the recruiter-recruit matrix stratified by age with the participant-contact matrix by age collected during the Dutch POLYMOD study (see Figure A5) [5, 6]. We used the Dutch POLYMOD data that was corrected for digit preference by participants for the age of contact persons, details on this correction can be found in Van de Kassteele, J., et al. [6].

Strong assortative mixing patterns by age were observed in both matrices. However, in our sampled recruiter-recruit matrix the younger age groups (below 20 years of age) were not represented. In the Dutch POLYMOD study these younger age groups were purposely oversampled to be able to analyse their contact patterns, as the hypothesis is that children play a central role in the transmission dynamics of influenza pandemics [6]. Children have frequent contact within their own groups and they have a wide range of contacts, therewith connecting all age groups [7].

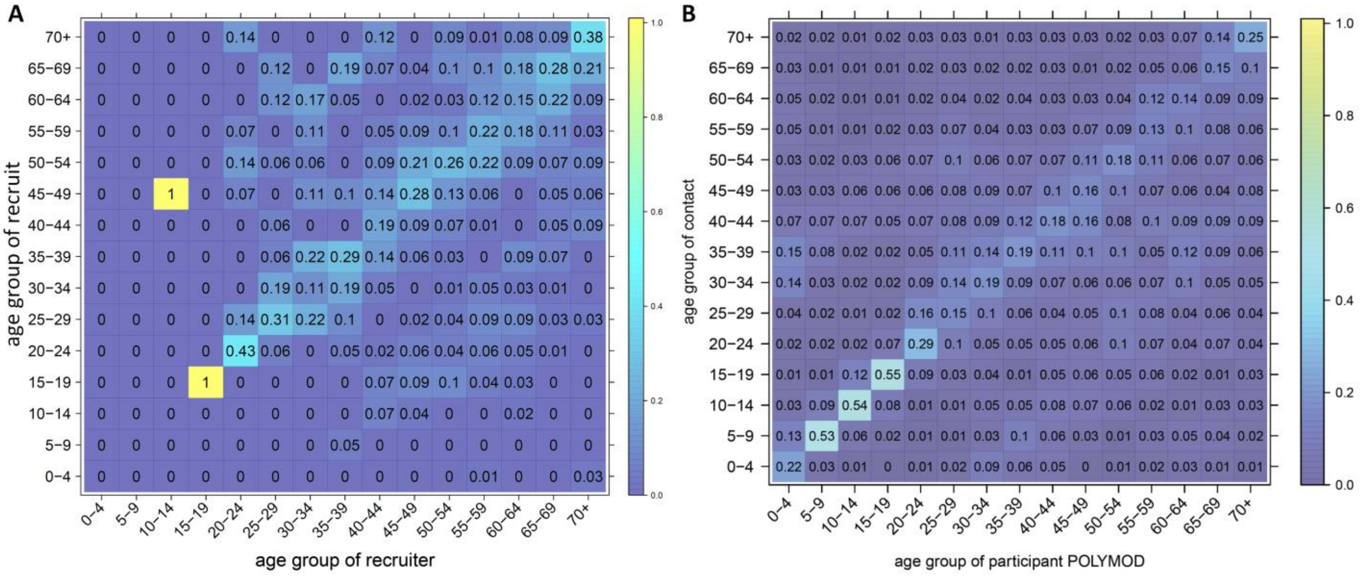

Figure A5. Comparison with the Dutch POLYMOD study. We compared the recruiter-recruit matrix stratified by age with the participant-contact matrix by age collected during the Dutch POLYMOD study [6]. (**A**) Sampled Recruiter–Recruit matrix, with respect to age; the values and colours indicate for each age group of recruiters the proportion of contact persons recruited. Thus, e.g. 0.43 for recruits between “20–24” indicates that from all recruits recruited by a recruiter from age group “20–24”, 43% was between 20–24 years. (**B**) POLYMOD participant–contact matrix, the values and colours indicate for each age group of POLYMOD participants the proportion of contact with persons of different age groups. POLYMOD had a correlation for age of 0.47 [0.45–0.48], p-value < 2.2e-16; df: 10186.

In Figure 2D (see main manuscript) we compared the number of contact persons reported at different locations by our participants, with the contacts reported at different locations in the Dutch POLYMOD study [6]. For this comparison the sample was weighted for the size of age groups in the POLYMOD study. The applied weights can be found in Table A3.

Table A3. Weighting sample for a comparison with POLYMOD

Age group	POLYMOD	SAMPLE[a]	Weight
0–39	439 (56.1%)	268 (17.2%)	3.27
40–49	76 (9.7%)	256 (16.4%)	0.59
50–64	132 (16.9%)	656 (42.1%)	0.40
65+	135 (17.3%)	379 (24.3%)	0.71

[a] The weights for age groups in our sample were obtained by dividing the POLYMOD proportion by the corresponding sample proportion.

3.2 Mixing by degree

We plotted the recruiter-recruit matrix by degree (see Figure A6). We observed random recruitment by degree between a recruiter and a recruit, which corresponds to the correlation coefficients for degree displayed in Table 3 in the main manuscript. As the dots clustered in the left corner, we looked more closely at the distribution up till an individual degree of 50. Furthermore, we plotted the distribution on a $\log_{10}$ scale, which also illustrated random mixing.

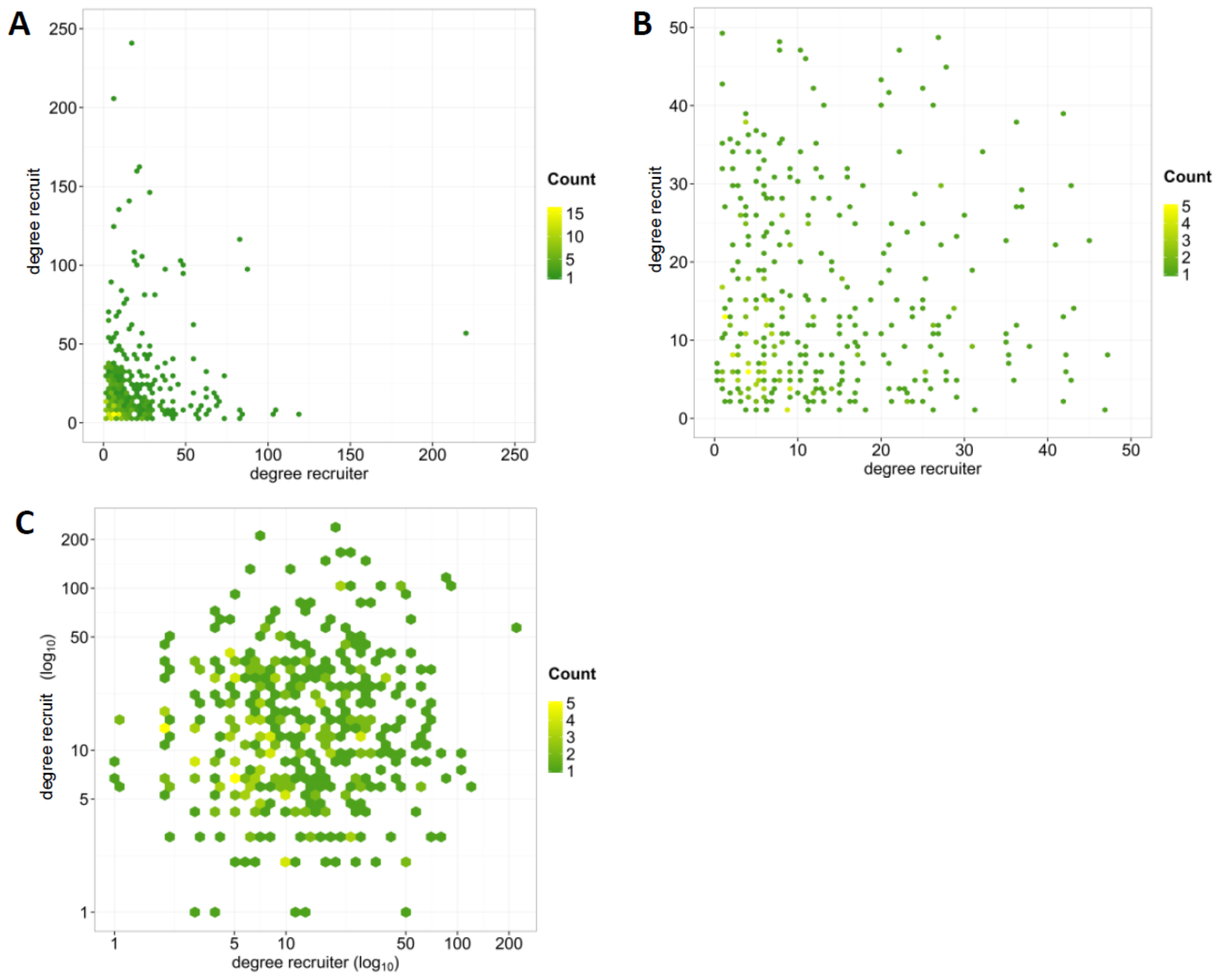

Figure A6. Recruiter degree versus recruit degree. (A) Untransformed. **(B)** Untransformed but axes limited to a degree of 50. **(C)** Log_{10} transformed.

3.3 Mixing by self-reported symptoms

Table A4 displays the absolute number of times a symptom was reported by participants. Runny or blocked nose was most frequently reported and vomiting the least frequent. The second column displays the number of recruiter-recruit pairs where only one of them reported the concerning symptom. Third column displays the number of pairs where both the recruiter and recruit reported the particular symptom. Runny and blocked nose was again most frequently reported, by either one of them or by both individuals in a pair.

Table A4. Self-reported symptoms and recruiter-recruit pairs with symptoms.

	$N_{\text{symptom reported}}$	$N_{\text{pairs only 1 had symptom}}$	$N_{\text{pairs both had symptom}}$
Fever	104	44	4
Chills	134	73	5
Runny or blocked nose	422	162	41
Earache	74	36	1
Sore throat	267	136	15
Cough	338	125	27
Stuffiness	146	68	4
Headache	360	153	36
Muscle / joint pain	292	149	30
Diarrhea	88	51	7
Vomit	26	14	0
Other symptoms	91	59	3

4. Distance between recruiter-recruit pairs

We investigated the distance between recruiters and recruits based on the provided 4-digit postal codes. The distribution of the distance between recruiter-recruit pairs was right-skewed (Figure A7). Based on the distribution in Figure A7, we categorised for Table 4 (see main manuscript) distance into three groups: same postal code, 1–10 km and > 10km. It appeared that for the first group (same postal code), recruiters invited slightly more similar aged recruits compared to the other two distance groups (see Figure A8). This is confirmed by the correlation coefficients for age in Table 4 in the main manuscript.

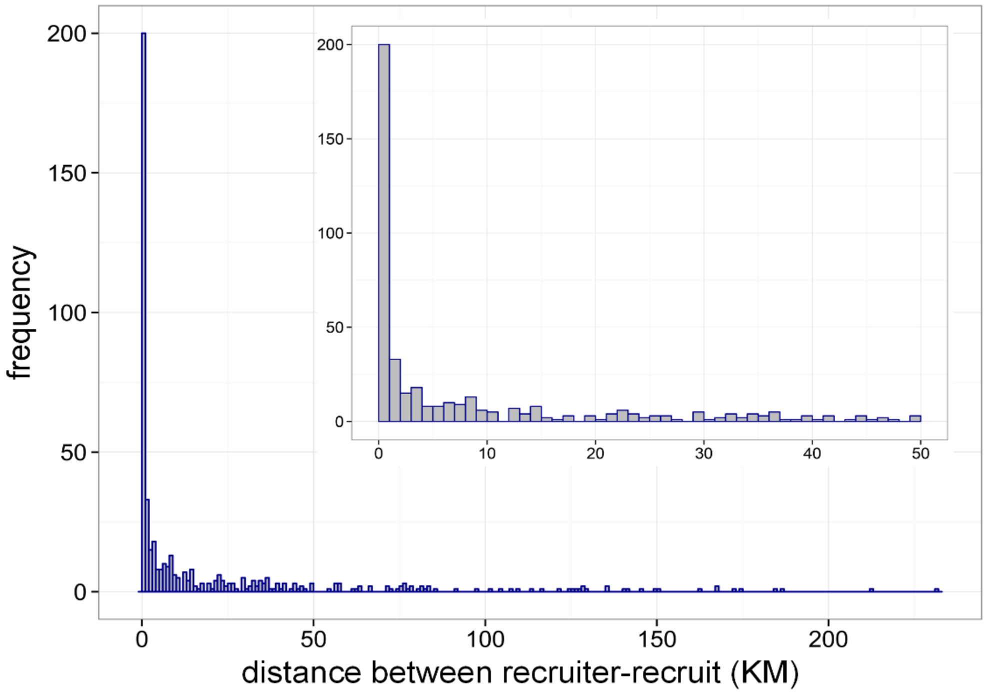

Figure A7. Histograms of distances between recruiter-recruit pairs in kilometres (km).

The mean distance between seeds (wave 0) and their recruits in wave 1 was higher than the mean distance between recruiter-recruit pairs in consequent waves. Figure A9 displays the distances for different link steps, as seen from the seed. Thus link step 1 is between seeds and their recruits in wave 1.

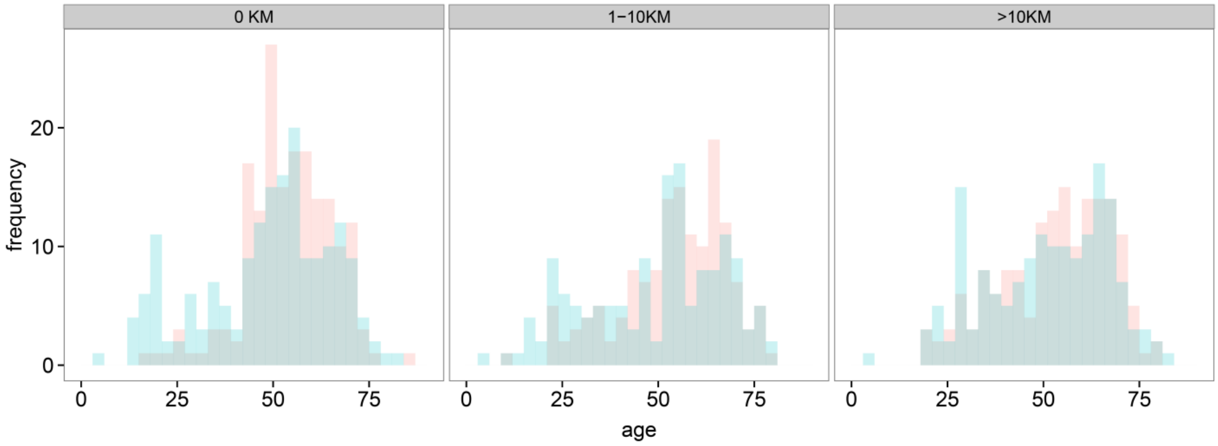

Figure A8. Age distributions of recruiters and recruits stratified by distance. Pink colour: recruiters; blue colour: recruits.

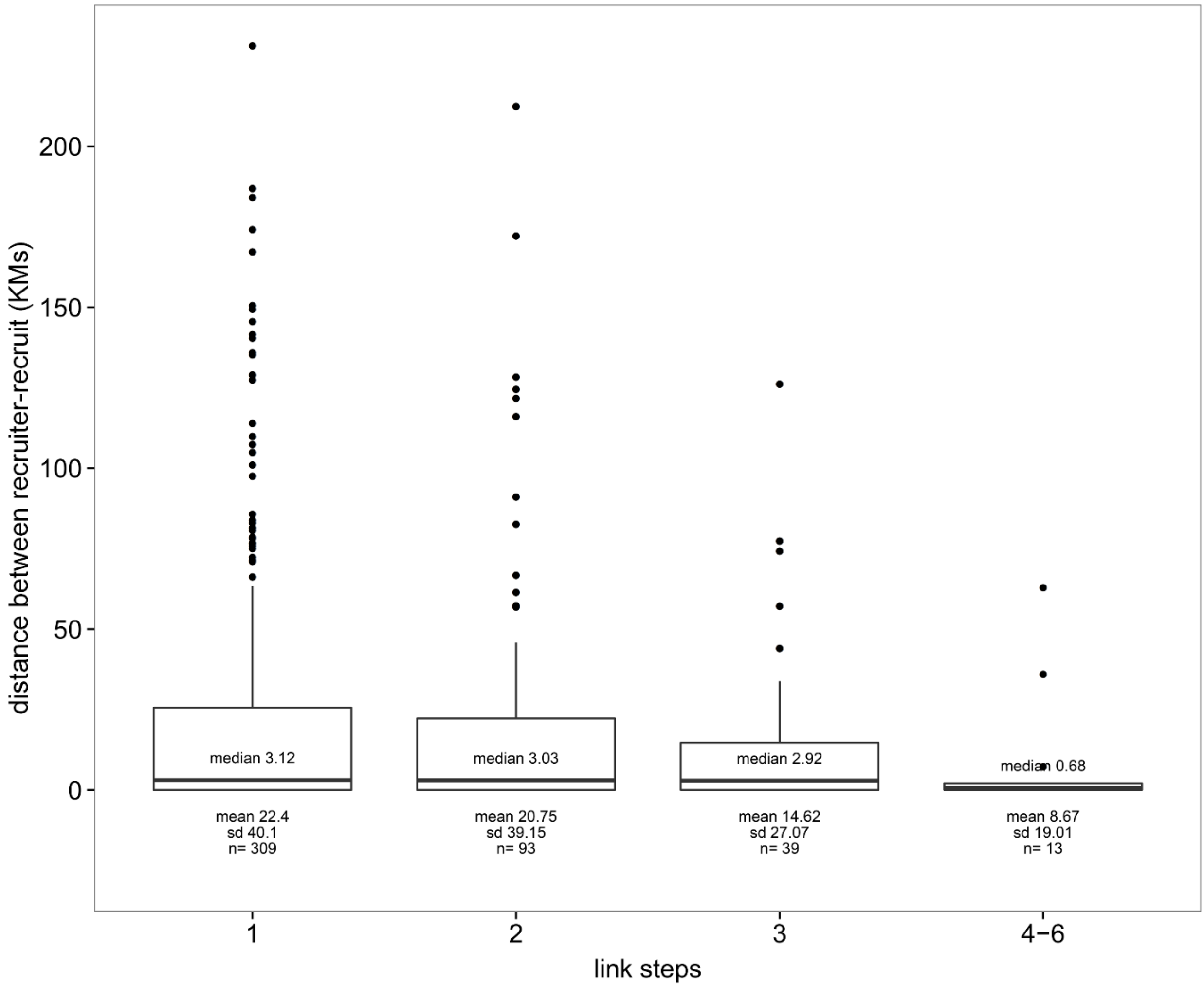

Figure A9. Distance between recruiter-recruit for different link steps. Link steps here indicate links steps as seen from the seed. Thus, link step '1' indicates the link between seeds and recruits-in-wave-1; link step 2 the link between recruiters-in-wave-1 and recruits-in-wave-2, and so on.

4.1 A logistic regression analysis

The Netherlands counts 12 provinces that represent the administrative layers between the national government and the local municipalities (i.e. subdivisions of a province). We categorised the Netherlands into four regions: North-Netherlands (Friesland, Groningen, Drenthe), Middle-Netherlands (Overijssel, Flevoland, Gelderland, Utrecht), West-Netherlands (North-Holland, South-Holland) and South-Netherlands (North-Brabant, Limburg, Zeeland).

Figure 4 demonstrated similar patterns between the recruitment trees (Figure 4A) and the commuting network (Figure 4B). Therefore, we investigated for Dutch participants the relationship between the geographical locations where a recruiter works and/or studies, and the location where their recruited contact person lives. We excluded participants living in Belgium. Home location was defined by the provided 4-digit postal code. The work location was defined by the city or village that was provided in the questionnaire.

Our goal was to quantify the extent to which a recruiter who lives in a certain region (four regions defined, see above) invites contact persons that live in the same municipality as the recruiter is working/studying.

We used a mixed effect logistic regression model to estimate the binary outcome:

- recruiter did *not* invite a recruit who lives in the same municipality as the recruiter is working or studying (0)
- recruiter invited a recruit who lives in the same municipality as the recruiter is working or studying (1)

This outcome variable was created through recoding:

- "municipality where recruiter works/studies" ≠ "municipality where his/her recruit lives" [1]
- "municipality where recruiter works/studies" = "municipality where his/her recruit lives" [2]

The log odds of the binary outcome was modelled as a linear combination of the variables "region of residence recruiter" (four regions) and "recruiter lives and works in same municipality" (binary: yes/no), with the region West-Netherlands and 'recruiter not working in the same municipality as he/she is living' as a reference group. The random intercept was provided by recruiter ID, to correct for differences between recruiters, e.g., in numbers of contact persons invited per recruiter and type of recruited contact persons.

Table A5 displays the 342 recruiters that were analysed stratified by outcome, location, and whether or not they live and work in the same municipality. For these 342 entries, the recruiter and his/her recruit:

- indicated that they lived in the Netherlands
- provided a work or study location in the questionnaire.

Table A5. Frequency table

**Province of residence**	**works and lives in same municipality**	**Recruiter *did not* invite recruit in same municipality as he/she is working/studying [1]**	**Recruiter invited recruit in same municipality as he/she is working/studying [2]**	**Total**
South-Netherlands	yes	15 (40.5%)	22 (59.5%)	37
	no	19 (79.2%)	5 (20.8%)	24
Middle-Netherlands	yes	33 (44.6%)	41 (55.4%)	74
	no	55 (90.2%)	6 (9.8%)	61
West-Netherlands	yes	28 (44.4%)	35 (55.6%)	63
	no	52 (96.3%)	2 (3.7%)	54
North-Netherlands	yes	5 (27.8%)	13 (72.2%)	18
	no	10 (90.9%)	1 (9.1%)	11
**Total recruiters***		**217**	**125**	**342**

*20 recruiters from Belgium were excluded. 144 recruiters indicated retirement, but a majority of them also indicated a location where they (still) work.

We used the fitted logistic regression model to estimate probabilities of the outcome (2) for the four regions in the Netherlands (i.e., predictions based on not knowing what recruiter ID is being predicted). Confidence intervals (95%) were calculated by both using fixed-effects uncertainty only, as well as by using fixed effects uncertainty + random effect variance.

Table A6 shows the output of the mixed effect logistic regression. The variable working and living in same municipality significantly influenced the outcome. Table A7 displays the estimated probabilities. Participants living in the North of the Netherlands had the highest probability, namely 0.774 [95% CI 0.433, 0.939], to invite a recruit in the municipality where they also work and live.

Table A6. Fixed effects

	Estimate[a]	SE	z value	Pr(>|z|)
Intercept	-3.632	0.749	-4.851	1.231E-06
Middle-Netherlands	0.288	0.521	0.553	5.802E-01
South-Netherlands	0.904	0.641	1.410	1.587E-01
North-Netherlands	0.982	0.819	1.198	2.308E-01
Work and live in same municipality	3.882	0.764	5.082	3.730E-07

[a] AIC 351.9; BIC 375.0; logLik -170.0; deviance 339.9; Random effect variance 3.319 (SE: 1.822); subjects: 223

Table A7. Estimated probability of inviting a contact person in the municipality where recruiter works or studies.

Region	Recruiter works/ studies and lives in same municipality?	Predict. Prob.	CI based on fixed-effects uncertainty ONLY		CI based on FE uncertainty + RE variance	
			2.5%	97.5%	2.5%	97.5%
West-Netherlands	no	0.026	0.006	0.106	0.001	0.576
Middle-Netherlands	no	0.034	0.009	0.121	0.001	0.633
South-Netherlands	no	0.061	0.016	0.209	0.001	0.764
North-Netherlands	no	0.066	0.012	0.290	0.001	0.801
West-Netherlands	yes	0.562	0.361	0.745	0.030	0.982
Middle-Netherlands	yes	0.631	0.439	0.789	0.040	0.986
South-Netherlands	yes	0.760	0.513	0.905	0.066	0.993
North-Netherlands	yes	0.774	0.433	0.939	0.062	0.994

References

1. Newman MEJ. Power laws, Pareto distributions and Zipf's law. Contemporary Physics. 2005;46:323-51.
2. Limpert E, Stahel WA, Abbt M. Log-normal distributions across the sciences: keys and clues. BioScience. 2001;51(5):341-52.
3. Hilbe JM. Alternative variance parameterizations: Poisson inverse Gaussian regression. Negative Binomial Regression. 2nd ed. New York: Cambridge University Press; 2011. p. 341-3.
4. Dean C, Lawless JF, Willmot GE. A mixed poisson-inverse-Gaussian regression model. The Canadian Journal of Statistics. 1989;17(2):171-81.
5. Mossong J, Hens N, Jit M, Beutels P, Auranen K, Mikolajczyk R et al. Social contacts and mixing patterns relevant to the spread of infectious diseases. PLoS medicine. 2008;5(3):e74.
6. Van de Kassteele J, Van Eijkeren J, Wallinga J. Efficient estimation of age-specific social contact rates between men and women. In preparation.
7. Mikolajczyk RT, Akmatov MK, Rastin S, Kretzschmar M. Social contacts of school children and the transmission of respiratory-spread pathogens. Epidemiology and infection. 2008;136(6):813-22.
